# Compositional zero-inflated network estimation for microbiome data

**DOI:** 10.1186/s12859-020-03911-w

**Published:** 2020-12-28

**Authors:** Min Jin Ha, Junghi Kim, Jessica Galloway-Peña, Kim-Anh Do, Christine B. Peterson

**Affiliations:** 1 Department of Biostatistics, University of Texas MD Anderson Cancer Center, 1400 Pressler St., Houston, TX USA; 2 Center for Devices and Radiological Health, U.S. Food and Drug Administration, 10903 New Hampshire Avenue, Silver Sp, MD USA; 3 Department of Veterinary Pathobiology, Texas A&M University, College Station, TX USA

**Keywords:** Microbiome, Network, Graphical model, Zero-inflation, Compositional data

## Abstract

**Background:**

The estimation of microbial networks can provide important insight into the ecological relationships among the organisms that comprise the microbiome. However, there are a number of critical statistical challenges in the inference of such networks from high-throughput data. Since the abundances in each sample are constrained to have a fixed sum and there is incomplete overlap in microbial populations across subjects, the data are both compositional and zero-inflated.

**Results:**

We propose the COmpositional Zero-Inflated Network Estimation (COZINE) method for inference of microbial networks which addresses these critical aspects of the data while maintaining computational scalability. COZINE relies on the multivariate Hurdle model to infer a sparse set of conditional dependencies which reflect not only relationships among the continuous values, but also among binary indicators of presence or absence and between the binary and continuous representations of the data. Our simulation results show that the proposed method is better able to capture various types of microbial relationships than existing approaches. We demonstrate the utility of the method with an application to understanding the oral microbiome network in a cohort of leukemic patients.

**Conclusions:**

Our proposed method addresses important challenges in microbiome network estimation, and can be effectively applied to discover various types of dependence relationships in microbial communities. The procedure we have developed, which we refer to as COZINE, is available online at https://github.com/MinJinHa/COZINE.

## Background

The communities of microorganisms living in and on the body, known as the human microbiome, have been shown to play an important role in both health and disease. In particular, the microbiome has been associated with conditions such as obesity [1], inflammatory bowel disease [2], colorectal cancer [3], and, more generally, with immune response and inflammation [4]. The role of the microbiome in modulating immune response has particular implications for cancer treatment, where characteristics of the patient’s microbiome have been associated with response to immunotherapy [5] and to the development of graft-versus-host-disease [6]. The community of microbial species constituting the microbiome is governed by a complex set of ecological interactions, and understanding these relationships may provide insight into intervention approaches aimed at restoring a healthy microbial community and reducing the risks of conditions associated with microbiome dysbiosis [7].

The most common strategy for profiling of microbial populations is sequencing of the variable region of the ribosomal 16S RNA gene. More recently, shotgun metagenomic sequencing has become available as an alternative. Using either approach, microbial abundances are quantified by grouping the observed sequences into operational taxonomic units (OTUs) based on their sequence similarity. Microbial associations can then be inferred based on the resulting abundance profiles. However, there are a number of challenges in determining the associations among microbial taxa. One challenge is the compositional nature of the data, which is due to both sampling and sequencing depth: the number of reads assigned to a given OTU can only be interpreted relative to the total number of reads obtained for the sample. Relying on standard Pearson or Spearman correlations among the microbial abundances can lead to spurious associations [8]. In addition, microbiome data tend to be highly zero-inflated, as many OTUs are observed in only a handful of samples. This means that assuming a standard distribution such as normal or Poisson on the OTU abundances may not be valid.

To address the challenge of inferring dependencies in microbiome data, a number of approaches have been developed to estimate sparse versions of the correlation or covariance matrix given compositional constraints. CCREPE [9] uses an ensemble approach to combine correlation and dissimilarity metrics into a single score, while SparCC [10] approximates the correlations among log-transformed abundances. More recently, computationally efficient methods using $\ell _1$ penalization, including CCLasso [11] and REBACCA [12], were proposed to obtain sparse estimates of the correlation structure. Finally, the composition-adjusted thresholding method [13] enables scalable estimation of a sparse version of the sample-centered log-ratio covariance matrix. These approaches focus on correlations, which are defined pairwise and may reflect indirect relationships among the covariates, which is a key limitation.

In contrast, graphical models focus on conditional dependencies between variables, and can be applied to obtain sparse networks reflecting direct relationships. In recent years, graphical models have become an important tool in learning biological networks from high-throughput data, with applications to a wide variety of data types including proteomics, transcriptomics, and metabolomics. The most popular method for graphical model estimation is the graphical lasso [14, 15], which uses a penalized approach to achieve sparse inference. The graphical lasso, however, assumes that the data come from a multivariate normal distribution, which is not a valid assumption for microbial abundances. The SPIEC-EASI procedure [16], which consists of a centered log-ratio transformation followed by either neighborhood selection or penalized maximum likelihood estimation, was proposed to adapt the graphical lasso to handle compositional data. More recently, methods have been developed to estimate a graphical model among a set of latent normal variables which are related to the observed counts via either a lognormal or logistic normal model [17, 18].

The network inference methods listed above are appropriate for compositional data; none, however, deal directly with zero-inflation, typically requiring that a small constant “pseudo-count” such as 0.5 or 1 be added to zero counts in the original data, and then treating these pseudo-counts as observed values. This approach has two major drawbacks, which are particularly critical when the proportion of 0s in the data is large. First, the assumptions made regarding how to “fix” the 0 count values can potentially have a large impact on downstream analysis. Second, the marginal distributions of each variable after transformation will still exhibit a peak corresponding to the spike at 0 in the original data, violating the assumption of normality and potentially leading to the discovery of spurious associations. In the context of single cell gene expression data, which is typically zero-inflated, a method based on the multivariate Hurdle model has been recently proposed [19] to estimate a network which reflects the relationships between both the presence or absence of a variable and the continuous representation of the variable. This model is not directly applicable to microbiome data, as it does not handle the fixed sum constraint. The Anets method [20] proposes getting rid of the fixed sum constraint by modeling only the associations between binary indicators of presence or absence. Since it ignores any differences in the non-zero abundance values, this approach cannot capture potentially important associations among common species. Similarly, the SPRING method [21], which estimates semi-parametric rank-based correlations, does not account for the magnitudes of the differences in abundance.

In the current work, we propose a novel procedure for sparse estimation of conditional dependencies between microbiome covariates which properly accounts for both the compositional and zero-inflated nature of the data. Specifically, we propose to transform only the non-zero values using the centered log-ratio transformation, while preserving the observed zeros. We then model the resulting values using the multivariate Hurdle model, and infer a sparse set of conditional dependencies which reflect not only relationships among the continuous values, but also among the binary values and between the binary and continuous representations of the data. We refer to this procedure as COZINE, for COmpositional Zero-Inflated Network Estimation.

## Methods

**Fig. 1:**
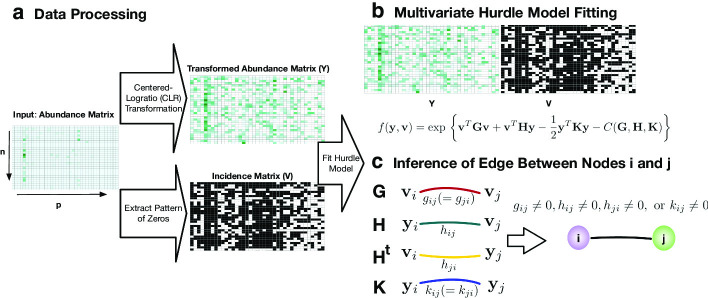
Schematic illustration of COZINE. **a** Using OTU abundance data matrix as an input data, the transformed abundance matrix ($\varvec{Y}$) and incidence matrix ($\varvec{V}$) are generated. **b** Hurdle Model is fitted on the combined dataset of $\varvec{Y}$ and $\varvec{V}$ using neighborhood selection approach with group-lasso penalty. **c** Network structure is constructed based on the structure of zeros in the parameter set, ${\mathbf {G}}$, ${\mathbf {H}}$ and ${\mathbf {K}}$

Using the $n \times p$ OTU abundance matrix as input, we aim to predict microbial relationships. As illustrated in Fig. 1, from the input data, we obtain two different representations: continuous values that represent the abundance of the microorganisms present in each sample, and binary data that represent presence or absence (Fig. 1a). Briefly, we fit the multivariate Hurdle model, which is comprised of a mixture of singular Gaussian distributions (Fig. 1b), and employ neighborhood selection with a group-lasso penalty to select conditional dependencies from the continuous abundance and the binary incidence data (Fig. 1c).

Our proposed COZINE method has three key innovative aspects: (1) we model conditional dependencies, as opposed to marginal dependencies, which can better capture complex forms of ecological interaction vs. pairwise correlations; (2) we explicitly model the excess of zeros in the OTU abundance data, avoiding the need to add a pseudo-count as in many existing methods; and (3) we bring together information on the binary absence–presence pattern and the continuous abundances to make inference on the ecological dependence structure. In the following subsections, we provide more details on the modeling approach.

### Network inference problem for compositional data

Let ${\varvec{W} }= (W_1,\ldots ,W_p)^{\mathrm {T}}$ with $W_j>0$ for all *j* be a vector of latent variables that represent the absolute abundances of *p* taxa, and denote the natural log transformed random vector of $\varvec{W}$ as ${\varvec{Z}}=(Z_1,\ldots ,Z_p)^{\mathrm {T}}=(\log W_1,\ldots , \log W_p)^{\mathrm {T}}$. Our aim is to construct the undirected graph on the random vector $\varvec{Z}$ that encodes the ecological relations of the *p* taxa. We represent the network of microbial associations as an undirected graph ${\mathcal {G}}=(U,E)$, where the set of nodes $U=\{1,\ldots ,p\}$ corresponds to the *p* taxa in $\varvec{Z}$, and the set of edges $E\subseteq U\times U$ includes undirected edges that represent co-occurrence (positive dependence) and mutual exclusion (negative dependence) relationships among the *p* taxa. Note that the random vectors $\varvec{W}$ and $\varvec{Z}$ are rarely observable due to limitations in sampling and sequencing. Instead, the data are typically expressed as random variables which correspond to proportional abundances:

1 $$\begin{aligned} X_j = \frac{W_j}{\sum \nolimits _{k\in U}W_k},\; {\text { for all }}\; j\in U. \end{aligned}$$

The random vector $\varvec{X}=(X_1,\ldots , X_p)^{\mathrm {T}}$ is a *composition* with non-negative components $X_1,\ldots ,X_p$ that are restricted to the simplex $\sum _{k\in U}X_k=1$. The positive random vector $\varvec{W}$ is called the basis, and represents the latent absolute abundances. The simplex constraint places a fundamental restriction on the degrees of freedom: the composition $\varvec{X}$ is essentially a $p-1$ dimensional random vector, and the components cannot be treated as independent random variables [22]. Our goal in this paper is to find the set of edges *E* that captures the underlying co-occurrence and mutual exclusion patterns in the compositional data of $\varvec{X}$.

### Handling of zero-inflation

An important feature of microbiome count data is the presence of a large number of zeros. A common strategy to handle these excess zeros is to add a small number called pseudo-count. This strategy is employed by many common methods that cannot directly handle zeros, including SparCC [10], CCLasso [11] and SPIEC-EASI [16]. Adding a pseudo-count to the zeros in microbiome data is simple, but the choice of value can influence downstream analysis and may neglect the information that some taxa are completely unobserved in the data. Also, the properties of the zero-inflation can be of intrinsic interest since co-occurrence and mutual exclusion relationships throughout the human microbiome would elucidate health status in human and their ecological relationships [23–25]. An intuitive approach to analyzing such zero-inflated count data is to view the data as arising from a mixture distribution of a point mass distribution at zero and a count distribution, such as Poisson [26]. With an unknown count distribution ${\mathcal {F}}$ and point mass distribution at zero ($I_0$) we assume that *W* follows a mixture distribution,

$$\begin{aligned} P(W = w) = \pi \;{\mathcal {F}} + (1-\pi ) \;I_0. \end{aligned}$$

with a mixture weight $\pi$. The model implies that zeros can arise from a count distribution, as well as the absence of a specific taxon in some subjects. Now we assume that the realization of *W*, $w \in \{0, 1, 2, \dots , \}$ where zero is considered for the support.

### Transformation of non-zero abundances

Now we describe the transformation of the non-zero count data, which is necessary to handle the compositional constraint. Key papers by Aitchison [22, 27] introduced the centered log-ratio (clr) transformation for studying compositional data. The clr transformation maps the compositional random vector $\varvec{X}$ from the unit simplex to a $p -1$ dimensional Euclidean space such that

$$\begin{aligned} \varvec{Y} = {\texttt {clr}}(\varvec{X}) = \left( {\mathbf {I}}_p - \frac{1}{p}{\mathbf {J}}_p \right) \ln \varvec{X}= { \mathbf {P}}\ln \varvec{X}, \end{aligned}$$

where ${\mathbf {I}}_p$ is the $p \times p$ identity matrix and ${\mathbf {J}}_p$ is a $p \times p$ matrix of 1s. $\varvec{Y}$ satisfies the zero sum constraint ${\mathbf {j}}^{\mathrm {T}}\varvec{Y}=0$, where ${\mathbf {j}}$ is the $p\times 1$ vector of 1s. However, the direct application of the clr formulation does not work for compositions with zero values. We modified the clr transformation to allow and keep the zero values in $\varvec{X}$ and $\varvec{Y}$. The vector $\varvec{V}\in \{0,1\}^p$ is defined through element-wise indicators of non-zero composition, i.e., $\varvec{V}=(V_1,\ldots ,V_p)^{\mathrm {T}}=(I(X_1\ne 0),\ldots I(X_p\ne 0))^{\mathrm {T}}$. The number of non-zero values in $\varvec{X}$ is denoted by $q={\mathbf {j}}^{\mathrm {T}}\varvec{V}$. Then the clr transformation of $\varvec{X}$ is: for all $j\in V$

2 $$\begin{aligned} Y_j = \left\{ \begin{array}{ll} \ln X_j - \frac{1}{q}\sum \limits {\{k:V_k=1\}} \ln X_k &{} {\text { if }}\quad V_j=1 \\ 0 &{} {\text { if }} \quad V_j=0.\\ \end{array} \right. \end{aligned}$$

We effectively remove the unit-sum constraint of the composition $\varvec{X}$ by transforming it to $\varvec{Y}$ with a zero-sum constraint, which is more tractable for use with standard statistical frameworks such as MANOVA, regression, and Gaussian graphical models [16, 28].

In summary, we first process the $n\times p$ OTU count data ${\mathbf {W}}$ into (1) an $n\times p$ binary matrix ${\mathbf {V}}$ by recording the zero (coded 0) and non-zero (coded 1) status of each element of ${\mathbf {W}}$, and (2) an $n\times p$ compositional abundance matrix ${\mathbf {X}}$ by applying Eq. (1) to each row of ${\mathbf {W}}$. To remove the row-wise unit sum constraint of the compositional data ${\mathbf {X}}$, we perform the clr transform in Eq. (2) to each row of ${\mathbf {X}}$ and the resulting transformed abundance matrix is denoted by ${\mathbf {Y}}$. Note that the zero values in the OTU table ${\mathbf {W}}$ are kept in the processed datasets ${\mathbf {V}}$ and ${\mathbf {Y}}$. The incidence matrix ${\mathbf {V}}$ and the clr-transformed abundance matrix ${\mathbf {Y}}$ are combined and used as the basis for network inference as described in the next section.

### Network inference

In order to infer a network using both the binary and continuous representations of the data, we rely on a multivariate Gaussian Hurdle model [19], which is based on the modification of the conditional Gaussian density [29–31] through excision of points in the support and assignment of positive masses to these points. Since our goal is to handle the zero-inflation of the *p* predictor variables, the excision points are all configurations of $\varvec{V}\in \{0,1\}^p$, each of which is assigned a positive density. Let ${\mathbf {y}}=(y_1,\ldots ,y_p)^{\mathrm {T}}$ denote a configuration of the clr-transformed random vector $\varvec{Y}$, and let ${\mathbf {v}}$ be a configuration of $\varvec{V}=\big (I(X_1\ne 0),\ldots ,I(X_p\ne 0)\big )^{\mathrm {T}}= \big (I(Y_1\ne 0),\ldots ,I(Y_p\ne 0)\big )^{\mathrm {T}}$, i.e., ${\mathbf {v}} = \big (I(y_1\ne 0),\ldots ,I(y_p\ne 0)\big )^{\mathrm {T}}$.

#### Binary–binary interactions

We assume $\varvec{V}$ to follow an Ising model with joint probability

3 $$\begin{aligned} p(\varvec{V};{\mathbf {G}}) \propto \exp \{\varvec{V}^{\mathrm {T}}{\mathbf {G}}\varvec{V}\}, \end{aligned}$$

where ${\mathbf {G}}$ is a $p\times p$ symmetric matrix of interaction parameters among the binary random variables $\{V_1,\ldots ,V_p\}$. The structure of zeroes in the off-diagonal elements of ${\mathbf {G}}$ encodes conditional independence relations among $\{V_1,\ldots , V_p\}$ [32].

#### Continuous–continuous interactions

Now suppose that the conditional distribution of $\varvec{Y}$ given $\varvec{V}={\mathbf {v}}$ is multivariate singular Gaussian distribution,

4 $$\begin{aligned} (\varvec{Y}|\varvec{V}={\mathbf {v}}) \sim N(\varvec{\mu }_{{\mathbf {v}}},{\mathbf {K}}_{{\mathbf {v}}}^{-1}), \end{aligned}$$

where we allow the normal distribution as singular and we have $2^p$ collection of mean vectors, $\{\varvec{\mu }_{\mathbf {v}}, {\mathbf {v}}\in \{0,1\}^p\}$ and precision matrices $\{{\mathbf {K}}_{\mathbf {v}}, {\mathbf {v}}\in \{0,1\}^p\}$. In our application, we assume *p* is large to be greater than the sample size and the models (3) and (4) involves $p\times p$ interaction matrix of $\varvec{V}$, *p*-dimensional mean vectors and $p\times p$ precision matrices for all possible $2^p$ configurations of $\varvec{V}$.

#### Binary–continuous interactions

We now reduce our model space by restricting ${\mathbf {K}} = {\mathbf {K}}_{\mathbf {v}}$, and introducing $p\times p$ interaction matrix ${\mathbf {H}}$ between ${\mathbf {v}}$ and ${\mathbf {y}}$ into the mean vector $\varvec{\mu }_{{\mathbf {v}}}$, $\varvec{\mu }_{\mathbf {v}} = ({\mathbf {I}}_{{\mathbf {v}}}{\mathbf {K}}{\mathbf {I}}_{{\mathbf {v}}})^{-} {\mathbf {H}}^T{\mathbf {v}}$ where ${\mathbf {I}}_{\mathbf {v}}$ is the $p\times p$ diagonal matrix with ${\mathbf {v}}$ in the diagonal elements, and $^-$ is the Moore–Penrose pseudoinverse. Using this re-parametrization, we can express the log-conditional density of $\varvec{Y}$ given $\varvec{V}={\mathbf {v}}$ as follows:

5 $$\begin{aligned} \log f({\mathbf {y}}|\varvec{V}={\mathbf {v}}) = {\mathbf {v}}^T{\mathbf {H}}{\mathbf {y}} - \frac{1}{2}{\mathbf {y}}^T{\mathbf {K}}{\mathbf {y}} - C({\mathbf {H}},{\mathbf {K}}). \end{aligned}$$

By combining Eqs. (3) and (5), the joint distribution of $\varvec{Y}$ and $\varvec{V}$ follows the multivariate Hurdle model [19]:

6 $$\begin{aligned} f(\mathbf {y, v}) = {\text {exp }}\bigg \{{\mathbf {v}}^{T}{\mathbf {G}}{\mathbf {v}} + {\mathbf {v}}^{T}{\mathbf {H}}{\mathbf {y}} - \frac{1}{2} {\mathbf {y}}^{T}{\mathbf {K}}{\mathbf {y}} - C'({\mathbf {G}}, {\mathbf {H}}, {\mathbf {K}})\bigg \}, \;\;\; {\mathbf {y}} \in \mathbb {R}^p, {\mathbf {v}}\in \{0,1\}^p, \end{aligned}$$

where $C'({\mathbf {G}}, {\mathbf {H}}, {\mathbf {K}})$ is a normalization constant, ${\mathbf {G}}$ and ${\mathbf {K}}$ are $p \times p$ symmetric matrices, and ${\mathbf {H}}$ is an arbitrary $p\times p$ matrix [19]. As seen in Eq. (6), the joint density is in an exponential family with three interaction matrices ${\mathbf {G}}, {\mathbf {H}}$, and ${\mathbf {K}}$ as natural parameters, and ${\mathbf {v}}{\mathbf {v}}^T, {\mathbf {v}}{\mathbf {y}}^T$ and ${\mathbf {y}}{\mathbf {y}}^T$ as sufficient statistics [19, 31].

#### Inference using neighborhood selection with group lasso penalty

The zero elements in the three interaction matrices, ${\mathbf {G}}=(g_{ij})$, ${\mathbf {H}}=(h_{ij})$ and ${\mathbf {K}}=(k_{ij})$ imply different types of conditional dependence relations between two nodes *i* and *j* in *U*:

1. $g_{ij}$ represents conditional dependencies for the presence–absence status of the two taxa,
2. $k_{ij}$ encodes conditional dependencies when the two taxa are observed,
3. $h_{ij}$ quantifies the mean level of abundance of taxa *i* when taxa *j* is present.

By the Hammersley–Clifford theorem [30], the conditional independence graph on the node $U=\{1,\ldots ,p\}$ has a missing edge between nodes *i* and *j*, $(i,j)\notin E$ if and only if the four possible interactions between *i* and *j* are all zero,

7 $$\begin{aligned} g_{ij}=h_{ij}=h_{ji}=k_{ij}=0. \end{aligned}$$

In other words, there is an edge $(i,j)\in E$ if and only if at least one of the four parameters, $g_{ij}$, $h_{ij}$, $h_{ji}$ and $k_{ij}$ are non-zero. For general exponential families, several methods have been proposed [33–35] to learn networks via a neighborhood selection approach that specifies the distribution of each node conditional on others. Following this approach, to estimate the parameters ${\mathbf {G}}$, ${\mathbf {H}}$, and ${\mathbf {K}}$ in Eq. (6), we learn the structure of zeros in the three matrices using the relatively tractable node-wise conditional distributions. For a fixed index *i*, define its complement $U^{[i]}= \{1,\dots ,p\} \backslash \{i\}$. Following [19], Eq. (6) can be rewritten in the form of $f_{i|U^{[i]}}({\mathbf {y}})$ for $i=1,\dots ,p$, where the vector of parameters describing the relation between nodes *i* and *j* are $\varvec{\theta }_{ij} = \left( g_{ij}, h_{ij}, h_{ji}, k_{ij} \right) ^{T}$ for $j \in U^{[i]}$. To impose sparsity on the graph structure, all four parameters in $\varvec{\theta }_{ij}$ must simultaneously be zero as in Eq. (7). To achieve this, for each node-wise regression, $f_{i|U^{[i]}}$, the group lasso penalty is imposed on $\varvec{\theta }_{i} = (\varvec{\theta }_{ij})_{j\in U^{[i]}}$ for a tuning parameter $\lambda \ge 0$:

$$\begin{aligned} P_{\lambda } (\varvec{\theta }_{i}) = \lambda \sum _{j \in U^{[i]}} \sqrt{\varvec{\theta }_{ij}^{T}\varvec{\theta }_{ij}} . \end{aligned}$$

Maximization of the penalized conditional log-likelihood function $f_{i|U^{[i]}}({\mathbf {y}}) - P_{\lambda } (\varvec{\theta }_i)$ can lead to a block-wise sparse solution, i.e. $\varvec{\theta }_{ij}=(0,0,0,0)^T$. We consider an edge (*i*, *j*) to be included in the final graph if any of the (*i*, *j*) or (*j*, *i*) entries in ${\mathbf {G}}$, ${\mathbf {H}}$, or ${\mathbf {K}}$ is non-zero.

## Results

### Simulation study

We performed simulation studies to compare the performance of the COZINE method with other approaches under simulation settings with various types of network topologies and parameters assumed. In particular, we applied SpiecEasi-MB (i.e., SPIEC-EASI using neighborhood selection) and SpiecEasi-Glasso (i.e., SPIEC-EASI using the graphical lasso) as implemented in the SpiecEasi package [16], as well as an Ising model fit using the neighborhood selection approach in the glmnet package. SpiecEasi does not consider the structure implied by the incidence matrix, as zeros in the input data are replaced with a small constant value, while the Ising model considers only the incidence data matrix ${\mathbf {V}}$, ignoring correlation patterns among abundances for taxa that are present.

To generate synthetic datasets, we considered two underlying topological structures: a band structure, specifically, an AR(1) graph; and scale-free networks generated according to the Barabasi–Albert model, BA(1), where at every time step one edge is added from a new node to an existing node that has larger number of connections [36]. The scale-free networks include hub nodes with high degree (number of connections), while AR(1) graphs consist of a big chain where each node has degree two. Given a topological structure, parametric assumptions in ${\mathbf {G}}$, ${\mathbf {H}}$ and ${\mathbf {K}}$ were varied by three settings (1) *G-minimal* network where the structure is only determined by the non-zero structure of ${\mathbf {G}}$, and ${\mathbf {H}}$ and ${\mathbf {K}}$ are set to be diagonal matrices; (2) *G–K* network where all edges are determined by ${\mathbf {G}}$ and part of the edges corresponding to the half of the nodes are also determined by ${\mathbf {K}}$; and (3) *G–H–K* network where all edges are determined by ${\mathbf {G}}$ and ${\mathbf {H}}$ but the structure of zeros in ${\mathbf {K}}$ determines the edges corresponding to the half of the nodes. For the two topological structures, AR(1) and BA(1), and the three parametric settings, we considered the 6 simulation settings as follows:

1. *G* *-minimal band network* where the structure of ${\mathbf {G}}$ follows AR(1), and ${\mathbf {H}}$ and ${\mathbf {K}}$ are diagonal matrices.
2. *G*–*Kband network* where the structure of ${\mathbf {G}}$ follows AR(1), nodes $p/2+1,\ldots ,p$ of ${\mathbf {K}}$ follow AR(1), and ${\mathbf {H}}$ is diagonal.
3. *G*–*H*–*Kband network* where the structures of ${\mathbf {G}}$ and ${\mathbf {H}}$ follow AR(1), and nodes $p/2+1,\ldots ,p$ of ${\mathbf {K}}$ follow AR(1).
4. *G*-*minimal scale-free network* where the structure of ${\mathbf {G}}$ follows BA(1), and ${\mathbf {H}}$ and ${\mathbf {K}}$ are diagonal.
5. *G*–*Kscale-free network* where the structure of ${\mathbf {G}}$ follows BA(1), nodes $p/2+1,\ldots ,p$ of ${\mathbf {K}}$ follow BA(1), and ${\mathbf {H}}$ is diagonal.
6. *G*–*H*–*Kscale-free network* where the structures of ${\mathbf {G}}$ and ${\mathbf {H}}$ follows BA(1), and nodes $p/2+1,\ldots ,p$ of ${\mathbf {K}}$ follows BA(1).

The resulting graphical structures of the first three follow a band structure, while the last three follow a scale-free network. Settings 1 and 4 consider *G*-minimal networks where ${\mathbf {H}}$ and ${\mathbf {K}}$ are diagonal matrices and only ${\mathbf {G}}$ contains non-zero off-diagonal elements. Settings 2 and 5 are *G*–*K* determined networks where the non-zero values of ${\mathbf {G}}$ and ${\mathbf {K}}$ imply the network structure, and ${\mathbf {H}}$ is a diagonal matrix. Finally, settings 3 and 6 are networks that are *G*–*H*–*K* determined where all the corresponding entries in each of the three matrices, ${\mathbf {G}}$, ${\mathbf {H}}$, and ${\mathbf {K}}$ for an edge are non-zero. In the first parametric setting, the *G*-minimal network, the Ising model is optimal for inference, while COZINE is over-parametrized by including ${\mathbf {H}}$ and ${\mathbf {K}}$. When ${\mathbf {H}}$ and ${\mathbf {K}}$ imply the graphical structure in the *G*–*K* and *G*–*H*–*K* settings, COZINE or SpiecEasi are the optimal choices. Thus, our simulation generation procedure is general enough to produce a wide range of simulation scenarios. The simulation studies for *K*-minimal and *H*–*K* networks are included in Section S1.1 of the Additional file 1. To generate edge values, the non-zero off-diagonal entries of ${\mathbf {G}}$, ${\mathbf {H}}$ and ${\mathbf {K}}$ were sampled from Unif $(-\,0.1,0.1)$ and the diagonal elements were set to the corresponding column sums plus 0.1 to ensure the matrices are positive definite. With $p=60$, we generated 200 samples from the multivariate hurdle model in Eq. (6) through Gibbs sampling with 2000 iterations after burn-in and 10% down-sampling [19]. Given the predefined structures of zeros in the $p\times p$ matrices, ${\mathbf {G}}$, ${\mathbf {H}}$, and ${\mathbf {K}}$, the compositional data $\{{\mathbf {X}}_k, k=1,\ldots n\}$ were generated as follows:

1. $\{\mathbf {Z}_i=(Z_{i1},\ldots ,Z_{ip})^{\mathrm {T}}, i=1,\ldots n\}$ are generated through Gibbs sampling from model (6) that was run for 2000 iterations after 1000 iterations of burn-in [19].
2. ${\mathbf {W}}_{i} = (W_{i1},\ldots ,W_{ip})^{\mathrm {T}}$ are obtained through the transformations $W_{ij}=e^{Z_{ij}}$.
3. ${\mathbf {X}}_{i}=(X_{i1},\ldots ,X_{ip})^{\mathrm {T}}$ are obtained by ${\mathbf {X}}_{ij} = \frac{W_{ij}}{\sum _{k=1}^pW_{ik}}$.

The final two steps ensure that the simulated data resemble real microbiome data, in that the non-zero values are highly skewed and have a unit sum constraint.

We assessed the accuracy in recovering the network structure in terms of the total number of true positive (*TP*), true negative (*TN*), false positive (*FP*), and false negative (*FN*) edges. We compute the true positive rate $TP/(TP+FN)$ and the false positive rate $FP/(TN+FP)$. Based on these measures, we plot the receiver operating characteristic (ROC) curves, along with the area under the curve (AUC) values, which reflect performance across a range of inferred network sizes. To obtain a balanced measure of accuracy for a single selected network, we rely on Matthew’s correlation coefficient (MCC), defined as

$$\begin{aligned} {\text {MCC}}=\frac{(TP\times TN)-(FP\times FN)}{\{(TP+FP)(TP+FN)(TN+FP)(TN+FN)\}^{1/2}}. \end{aligned}$$

The MCC ranges from $-\,1$ (total disagreement) to 1 (perfect agreement).

**Fig. 2:**
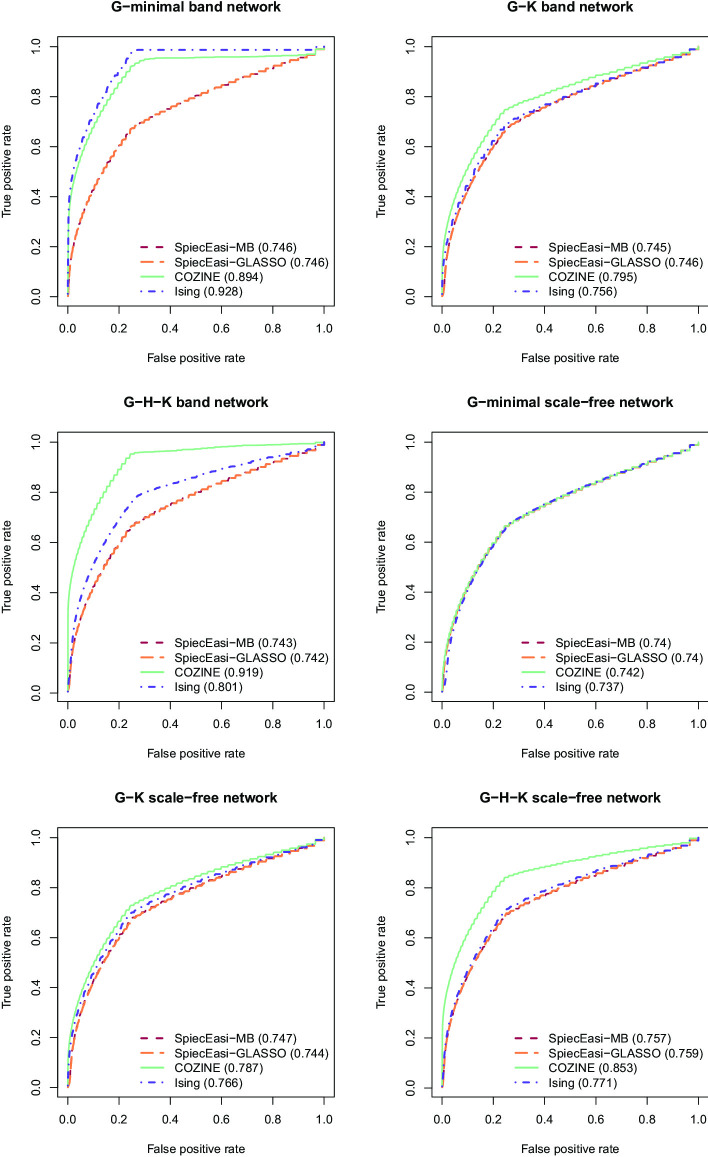
Performance comparison on simulated data. ROC curves and AUC values for SpiecEasi-MB, SpiecEasi-GLASSO, COZINE, and Ising model under the 6 different simulation scenarios

**Fig. 3:**
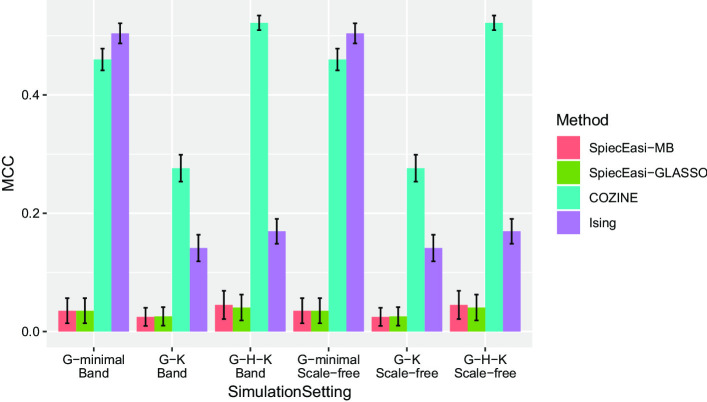
MCC values across simulation settings

Based on 25 synthetic datasets with $n=200$ and $p=60$, we evaluated the performance of the COZINE method. Overall, the COZINE method showed the highest accuracy in terms of the ROC analysis across all simulation settings, except for the *G*-minimal band network scenario where the Ising model performs the best (Fig. 2). This is along the expected lines as the structural information in this network is encoded only in the parameter ${\mathbf {G}}$. In this setting, COZINE had much better performance compared to the two SPIEC-EASI methods that ignore the zero values in the data. When the underlying true network structure became more complex, changing from *G*-minimal band network to *G*-minimal scale-free network, COZINE performed better than the Ising model. When the network structure also quantifies the mean levels of the abundance when other species are present (non-zero off-diagonal entries of ${\mathbf {H}}$) in the *G*–*H*–*K* complete band and scale-free network scenarios, the COZINE method gained the highest structural accuracy compared to all other three methods. We also compared the MCC values across the 6 simulation settings (Fig. 3). MCC values were positive across all methods and simulation settings, however, both of the SPIEC-EASI methods result in much lower MCC values than the COZINE and Ising methods. As expected, the Ising model provides better MCC values for the *G*-minimal scenarios, and COZINE performs the best in the *G*–*K* and *G*–*H*–*K* cases regardless of the network topology. Additional simulation results for *K*-minimal and *H*–*K* networks, as well as high-dimensional networks with $p=1000$ nodes, are provided in Section S1 of the Additional file 1.

### Application to oral microbiome data

The microbiome plays a critical role in human health, immunity, and disease, and its composition is governed in part by complex ecological interactions. We illustrate the proposed COZINE method to infer a microbial co-occurrence network from 86 oral samples obtained from newly diagnosed adult acute myeloid leukemia (AML) patients undergoing induction chemotherapy (IC) at the University of Texas MD Anderson Cancer Center, measured at baseline before the start of IC. Many AML patients will go on to develop oral or dental complications from their cancer treatment, and the composition of the oral microbiome plays a role in determining this risk. In particular, the oral microbial composition has been shown to be associated with the development of oral mucositis, which is characterized by ulcerative lesions in the mouth, in hematopoietic stem cell transplantation patients with hematologic malignancies [37, 38]. Microbiome risk factors have also been associated with the development of oral candidiasis, which is an infection of the oral cavity, during cancer chemotherapy [39]. Characterizing the ecological relationships in the oral microbiome of AML patients is therefore of interest to understand potential underpinnings of subsequent oral microbiome dysbiosis. For additional details on the cohort and sample processing, see “Appendix”.

**Fig. 4:**
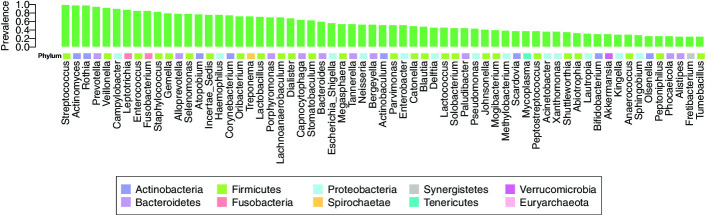
Prevalence of oral genera across samples. Prevalence for 63 genera in oral microbiome samples

We analyzed the microbial composition data at the genus level by summation of the OTU counts within genera. Analysis results at the OTU level are included in Section S3 of the Additional file 1. We screened the genera to those with prevalence (the proportion of non-zero abundances across samples) of at least 25%, resulting in 63 genera. Figure 4 displays prevalence for the 63 genera identified, which are included as nodes in the network inference.

**Fig. 5:**
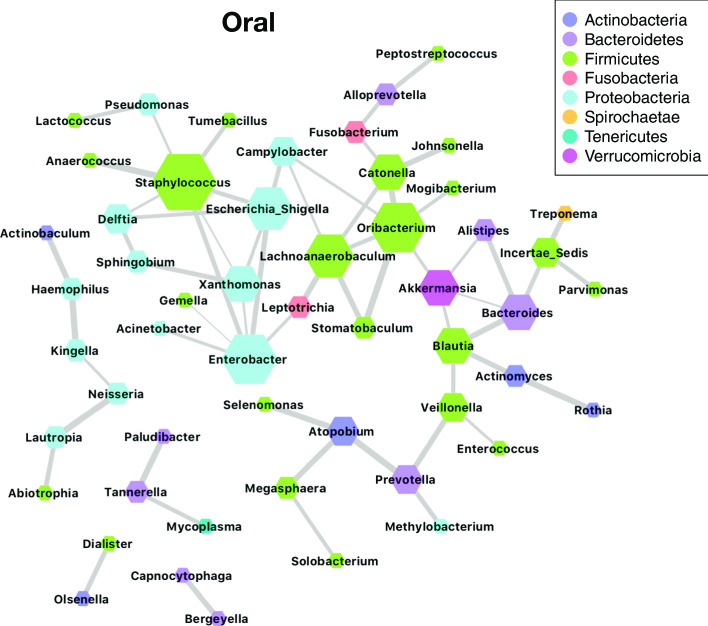
Co-occurrence network for oral samples. Nodes are weighted by degrees and edges are weighted by stability

The COZINE method took 184 s on a Linux server (2.93 GHz; 96 GB RAM) for the 63 genera. In the resulting network, nodes correspond to the bacterial genera, with each edge representing a dependence relation. We found 59 edges in the oral microbial network. To assess the stability of the edges in the network, we generated 100 bootstrap samples, and applied the COZINE method on each of the bootstrap samples. The stability of each edge is defined by the proportion of bootstrap samples where the resulting networks include the edge. The first quantile of the stability values was greater than 0.75, which indicates that most edges appeared in more than 75 bootstrap networks, and are hence robust. Figure 5 shows the resulting network, where the nodes were weighted by degree (number of incident edges), and the edges were weighted by stability. Table 1 provides a detailed listing of the most stable edges identified, along with their stability score and weight.

**Table 1:** Stability and weight for edges with stability $\ge$ 0.9

Node 1	Node 2	Stability	Weight
Stomatobaculum	Oribacterium	1.00	0.80
Atopobium	Prevotella	0.99	0.39
Neisseria	Lautropia	0.99	0.4
Kingella	Haemophilus	0.99	0.27
Anaerococcus	Staphylococcus	0.99	0.37
Paludibacter	Tannerella	0.97	0.47
Actinomyces	Rothia	0.97	− 0.05
Catonella	Oribacterium	0.95	0.35
Bacteroides	Blautia	0.94	0.33
Atopobium	Megasphaera	0.94	0.43
Atopobium	Selenomonas	0.94	0.13
Actinobaculum	Haemophilus	0.94	0.05
Leptotrichia	Lachnoanaerobaculum	0.93	0.29
Catonella	Johnsonella	0.92	0.32
Stomatobaculum	Lachnoanaerobaculum	0.92	0.44
Capnocytophaga	Bergeyella	0.92	0.01
Prevotella	Veillonella	0.92	0.29
Incertae Sedis	Treponema	0.91	0.11
Escherichia Shigella	Enterobacter	0.91	0.32
Actinomyces	Blautia	0.90	− 0.04

As seen in Figs. 4 and 5, genera belonging to the phyla Firmicutes and Proteobacteria were highly prevalent in the oral samples from AML patients analyzed here. These genera showed a number of interactions among themselves and also with genera from other phyla, playing a role as hubs within the inferred network. The major role of Firmicutes and Proteobacteria in the oral microbiome has previously been noted in the Human Microbiome Project (HMP) cohort [9], and several of the highly stable relationships among genera found in our network are supported in the literature. In particular, the pairs of genera *Leptotrichia*–*Lachnoanaerobaculum* and *Catonella*–*Oribacterium* were shown to co-occur in patients with oral cancer [40], based on pairwise Pearson correlation. Several relationships found among genera in the AML patient cohort using COZINE have also been observed among healthy individuals. The pairs *Neisseria*–*Lautropia* and *Prevotella*–*Veillonella* were found to be correlated in salivary samples from healthy individuals with different diet types [41]. *Actinomyces*–*Rothia* and *Lautropia*–*Neisseria* were shown to co-localize in dental plaque samples [42]. Finally, *Prevotella* has also been shown to co-occur with *Atopobium* in oral samples of healthy subjects [43]. We also found *Staphylococcus* to be the most highly connected genus in our inferred graph structure. *Staphylococcus* bacteria can cause many types of infections including skin infections, pneumonia, and bloodstream infections in cancer patients, which are associated with higher mortality than those caused by other pathogens [44]. This offers new insights in oral dysbiosis during cancer treatment and in hospitalized patients. However, we have a unique cohort of subjects undergoing cancer treatment, which are receiving chemotherapy, antimicrobial therapy, and experience severe cases of mucositis which may result in a very different oral microbial environment and the connectivity. Moreover, the HMP data has shown that the biogeography of the oral cavity is complex, thus our buccal swabs may exhibit different key organisms and relationships than dental plaque or sputum. Thus, we do not necessarily expect our data to overlap with findings from published cohorts. In addition to recapitulating known dependencies, our results also uncover a number of novel relationships, providing insight into the complex web of interactions within the oral microbiome in our patient cohort.

#### Assortative network structure

We investigate the tendency of taxa which occur in the same branch of the taxonomic tree to be linked within co-occurrence networks, a pattern which has been noted in previous works on microbial network inference [9, 16]. Since our network was constructed using quantifications at the genus level, in examining the assortativity we considered classifications at higher taxonomic levels, specifically, at the kingdom, phylum, class, and family levels. For each taxonomic classification, we calibrated the assortative coefficient [45] under the hypothesis that assortative mixing by taxonomy would break the network up into separate communities.

The assortative coefficient *r* ranges from $-\,1$ to 1 [45]. When the network has no assortative mixing (independent), $r=0$, and $r=1$ if there is perfect assortative mixing, i.e., all edges connect nodes within the same taxonomic grouping. When every edge connects two nodes from different taxonomic groups (completely dissortative), *r* can be any negative value between $-\,1$ and 0. When the network is completely random, the coefficient is closer to that of a dissortative network as the number of taxonomic classifications increases. Therefore, to evaluate the significance of the deviation from random mixing for a given network structure, and the number and proportions of taxonomic groups, we generate *r* under the null hypothesis of random mixing by permuting the taxonomic assignment of the nodes, and define a *p* value as the proportion of the values generated under the random mixing that are greater than the observed one.

The estimated network obtained from COZINE had assortativity coefficients (*p* values evaluated from 100,000 permutations) of 0.26 (0.0001), 0.22 (< 1e–05), 0.15 (< 1e–05) and 0.1 (< 1e–05) for phylum, class, order, and family, respectively, implying that the network is much more strongly assortative by taxonomic classification than one would expect on the basis of random chance. We estimated microbial networks using the Ising and SPIEC-EASI approaches, and compared the assortative coefficients with COZINE (Table 2). The networks from the Ising, SpiecEasi-MB, and SpiecEasi-GLASSO had 117, 61 and 27 edges, respectively. The networks constructed from COZINE showed the most significant assortative mixing across all phylogenetic classifications.

**Table 2:** Assortativity coefficient (*p* values from 100,000 permutations)

Level	COZINE	SpiecEasi (MB)	SpiecEasi (GLASSO)	Ising
Phylum	0.26 (0.0001)	0.22 (0.0002)	0.29 (0.0013)	0.14 (0.0003)
Class	0.22 (< 1e–05)	0.16 (< 1e–05)	0.20 (3e–04)	0.06 (0.0019)
Order	0.15 (< 1e–05)	0.08 (0.0014)	0.11 (0.0029)	0.04 (0.0044)
Family	0.10 (< 1e–05)	0.10 (1e–05)	0.15 (1e–05)	0.06 (2e–05)

Section S2 of the Additional file 1 includes additional evaluation of performance of 6 different methods, 4 partial correlation based methods (COZINE, Ising, SpiecEasi-GLASSO and SpiecEasi-MB), and two marginal correlation-based methods (SparCC [15] and CCLasso [11]), in terms of stability of edges and assortativity of the network topologies.

## Discussion

We have developed a novel method for the discovery of various types of interactions within microbial communities based on high-throughput profiling. Our proposed COZINE method handles data that is both compositional and zero-inflated, making it well-designed for application to microbiome data. Unlike existing methods for microbial network inference, we allow interactions that capture dependence between the presence or absence of features, between presence or absence of one feature and abundance of another, and between the continuous abundances for features that are present.

We illustrate the application of our method in both simulation settings with various network structures, and in an application to oral microbiome samples collected from a cohort of 86 AML patients. In simulation settings, our method achieves better accuracy in recovering the true network structures in all settings except the simplest scenarios where the only true dependencies are between the binary presence or abundance of a feature.

COZINE learns the network topology from both the binary incidence matrix, representing presence or absence of microbiome features across samples, and the transformed abundance matrix, using a modeling framework that includes three types of edges, binary–binary relationships in ${\mathbf {G}}$, binary–continuous relationships in ${\mathbf {H}}$, and continuous–continuous relationships in ${\mathbf {K}}$. We compared our method with the SpiecEasi and Ising methods that only model ${\mathbf {K}}$ and ${\mathbf {G}}$, respectively. We assessed the COZINE method in different simulation scenarios including both low and high dimensional settings and different levels of sparsity of the data, generated under networks with different topological properties, AR(1) and BA(1). Since the methodologies compared with COZINE are based on different modeling frameworks, we considered various parametric assumptions for ${\mathbf {G}}$, ${\mathbf {H}}$ and ${\mathbf {K}}$ in the data generation procedure, specifically, *G*-minimal, *K*-minimal, *G*–*K*, *H*–*K*, and *G*–*H*–*K* networks. Across all simulation settings except for the *G*-minimal setting, COZINE showed better performance than the SpiecEasi and Ising approaches in estimating the graphical structure. The use of a group-lasso penalty in COZINE that induces the same sparsity across the three types of relations was shown to perform well even when the models are mis-specified. However, in the cases where the underlying networks for binary–binary, binary–continuous and continuous–continuous relations are significantly different, i.e., at least one of the parameters, $g_{ij}$ , $h_{ji}$, $h_{ij}$ and $k_{ij}$ for an edge $i-j$ have zero values or very small effect sizes, then the implicit assumption that the four parameters have similar effect sizes is violated and may result in a loss of accuracy, mostly false negatives, in network estimation. Extensions that allow different structures of zeros in ${\mathbf {G}}$, ${\mathbf {H}}$ and ${\mathbf {K}}$ would be useful in understanding different microbial mechanisms encoded in interactions among binary and continuous representations of microbiome data.

We applied COZINE to our case study data defined at the OTU level (Additional file 1: Section S3). The data include 2029 OTUs and show a high level of sparsity, with a proportion of zero values of 95%. Our method took 3.03 h on a Linux server (2.93 GHz, 96 GB RAM) to run on this data. Using our penalized node-wise regression framework with the group-lasso penalty, we can directly estimate edges in the network by reading the zero-structure in ${\mathbf {G}}$, ${\mathbf {H}}$ or ${\mathbf {K}}$. The bootstrap procedure can be used for evaluating the stability of the estimated edges, but is not required for network inference. Utilizing our node-wise neighborhood selection procedure, the computational efficiency can be significantly improved by parallel computing by running each univariate regression in a single computing node. The reported run times were measured using only 2 computing nodes to learn all the 2029 node-wise regressions, which is the default setting for the COZINE function in our R package. The bootstrap procedure can also proceed in parallel for each bootstrap sample, enabling scalability to settings with large *p*.

## Conclusions

Our method is generally applicable to any data which exhibit both zero inflation and a fixed-sum constraint per sample. In our real data application to oral microbiome profiles from a cohort of 86 AML patients, we infer a sparse network where the majority of edges exhibit high stability and the topological structure showed high correlation with taxonomic tree. We observed that the microbial network inferred by COZINE identified known relations established in previous studies. *Firmicutes* and *Proteobacteria* had a dominant role in our microbial network, which aligns with findings for oral samples in the Human Microbiome Project. Our results both confirm co-occurrence relationships previously reported in the literature, and identify potentially interesting new aspects of the microbial interaction network. For example, we found *Staphylococcus* to be the most highly connected genus in our inferred graph structure. *Staphylococcus* bacteria can cause many types of infections including skin infections and pneumonia, and bloodstream infections by *Staphylococcus* in cancer patients are associated with higher mortality than those caused by other pathogens [44]. Future studies that investigate longitudinal changes in the topological structure of the microbial network during and after chemotherapy could inform interventional strategies aimed at shifting the oral microbiome towards a healthier state. The code implementing our method is freely available online at https://github.com/MinJinHa/COZINE.

## Supplementary Information

**Additional file 1:** Additional simulation results (section S1) and real data application results (section S2 and S3).

## Data Availability

16S rRNA V4 region sequences for the case study were obtained from the NCBI Sequence Read Archive http://www.ncbi.nlm.nih.gov/sra using the BioProject IDs PRJNA352060 and PRJNA526551. The code implementing our method is freely available online at https://github.com/MinJinHa/COZINE.

## Appendix

### Data processing

16S rRNA V4 region sequences for the case study were obtained from the NCBI Sequence Read Archive http://www.ncbi.nlm.nih.gov/sra using the BioProject IDs PRJNA352060 and PRJNA526551. 16S rDNA sequences were derived from study subjects consisting of 86 newly diagnosed adult AML patients undergoing IC at MD Anderson Cancer Center in Houston, TX from September 2013 to August 2015, and data from subsets of this cohort have been previously analyzed by Galloway-Peña et al. [46]. The real data is performed using sequencing of the 16S rRNA V4 hypervariable region. This is one of the most common regions sequenced when performing marker gene analysis for microbiome data. Unfortunately, the 16S rRNA V4 hypervariable region can only bin amplicon sequences to the genus level with utmost confidence. Many amplicons cannot be differentiated to the species level. See the methods detailed therein for a detailed description of the data processing.

We built the microbial composition data at the genera level by summing over all OTU counts within genera. The $\texttt {clr}$-transformed abundance matrix ${\mathbf {Y}}=(y_{ij})$ was conditionally centered given the incidence data ${\mathbf {V}}=(v_{ij})$ before fitting the network model:

$$\begin{aligned} \tilde{y}_{ij}= \left\{ \begin{array}{ll} y_{ij}-\bar{y_j}^+&{} {\text { if }} \quad v_{ij}=1 \\ 0 &{} {\text { if }} \quad v_{ij}=0,\\ \end{array} \right. \end{aligned}$$

where $\bar{y_j}^+$ is the average in a genus over the non-zero abundances, to make $V_j$ and $Y_j$ marginally orthogonal and speed up the convergence of the optimization [19].

### Network assortativity

Given an estimated microbiome network, ${\mathcal {G}}=(U,E)$, we consider assortative mixing [45] according to taxonomic classification at each level of the tree, e.g., kingdom, phylum, class, order, family, and genus. At a given level of the tree, the taxonomic grouping can be considered as an attribute of the node. For example, in our data analysis, we built the network at the genus level, and each genus is classified into one of the categories at the phylum level. Our goal is to quantify the level and its significance of the preference of linking to nodes with same ancestors.

Given a phylogenetic classification and a microbiome network, we define $e_{ij}$ as the proportion of edges in the network that connect a node of ancestor *i* to a node of ancestor *j*. As we consider undirected microbial network, $e_{ij}=e_{ji}$ and $\sum _{ij} e_{ij} = 1$. We further define $\sum _{i}e_{ij} =a_i$ as the proportion of edges that are attached to nodes of type *i*. Then assortativity coefficient [45] is:

$$\begin{aligned} r=\frac{\sum _{i}e_{ii}-\sum _{i}a_i^2}{1-\sum _{i}a_i^2}, \end{aligned}$$

where $r=0$ when the network has no assortative mixing ($e_{ii}=a_i^2$), and $r=1$ when there is perfect assortative mixing ($\sum _{i}e_{ii}=1$). If every edge connects two nodes of different ancestors (completely disassortative), then *r* is a negative value in the range $-1<r<0$ :

$$\begin{aligned} r_{min} = -\frac{\sum _{i}a_i^2}{1-\sum _ia_i^2}, \end{aligned}$$

with $\sum _ie_{ii}=0$. A random mixed network is naturally closer to a dissortative network as the number of ancestor categories increases [45]. Therefore, for evaluating if the assortative mixing of a network by phylogenetic classification is significantly deviated from the random mixing, we use permutation approach to produce the null distribution of the assortativity coefficient *r* given a fixed network topological structure and the number and sizes of ancestor categories. We quantified the significance of the observed assortativity coefficient by comparing it with the values obtained from random permutation of the node attributes: a *p* value for the coefficient is calculated by proportion of the random values that are greater than the observed.

## Abbreviations

OTU: Operational taxonomic unit
clr: Centered log-ratio
MANOVA: Multivariate analysis of variance
AR: Autoregressive
BA: Barabasi–Albert
TP: True positive
TN: True negative
FP: False positive
FN: False negative
ROC: Receiver operating characteristic
AUC: Area under the curve
MCC: Matthew’s correlation coefficient
AML: Acute myeloid leukemia
IC: Induction chemotherapy
HMP: Human Microbiome project
