# Supplementary material for: Compositional zero-inflated network estimation for microbiome data
Source: BMC Bioinformatics. 2020 Dec 28;21(Suppl 21):581. doi: 10.1186/s12859-020-03911-w (PMC7768662; doi:10.1186/s12859-020-03911-w)
Supplement: Supplementary file 1 — Additional file 1: Additional simulation results (section S1) and real data application results (section S2 and S3). [file 12859_2020_3911_MOESM1_ESM.pdf]

Supplementary Materials for "Compositional zero-inflated network estimation for microbiome data"

Min Jin Ha, Junghi Kim, Jessica Galloway-Peña, Kim-Anh Do,
and Christine B. Peterson

S1 Additional simulations

S1.1 K-minimal and H-K networks

Here we provide additional simulation studies for AR(1) graphs: (1) *K-minimal* network where the structure is only determined by the non-zero structure of $\mathbf{K}$, and $\mathbf{G}$ and $\mathbf{H}$ are set to be diagonal matrices; and (2) *H-K network* where all true edges have a nonzero entry in $\mathbf{K}$, with half of those edges represented in $\mathbf{H}$ as well, and $\mathbf{G}$ is set to be a diagonal matrix. All other steps of the simulation setup were kept the same as in the G-minimal, G-K and G-H-K simulations as described in the simulation section.

Based on 25 synthetic datasets, we compared the SpiecEasi-MB, SpiecEasi-GLASSO, COZINE and Ising methods using ROC analysis (Figure S1). COZINE outperformed all other methods for both simulation settings. When the network structure is solely determined by continuous interactions (K-minimal network), SpiecEasi is in theory the optimal choice, as the COZINE model is over-parametrized by including $\mathbf{G}$ and $\mathbf{H}$. However, COZINE achieved better accuracy in estimating the network structure than both approaches of SpiecEasi. This result suggests that the group lasso penalty utilized by COZINE that induces the same inclusion status for an edge across g_{ij}, h_{ij}, h_{ji} and k_{ij} can correctly detect edges encoded in any combination of the four parameters. When the network structure also encodes a dependence of the mean levels of abundance on the presence of other species (H-K network), COZINE achieves the highest accuracy across all four methods.

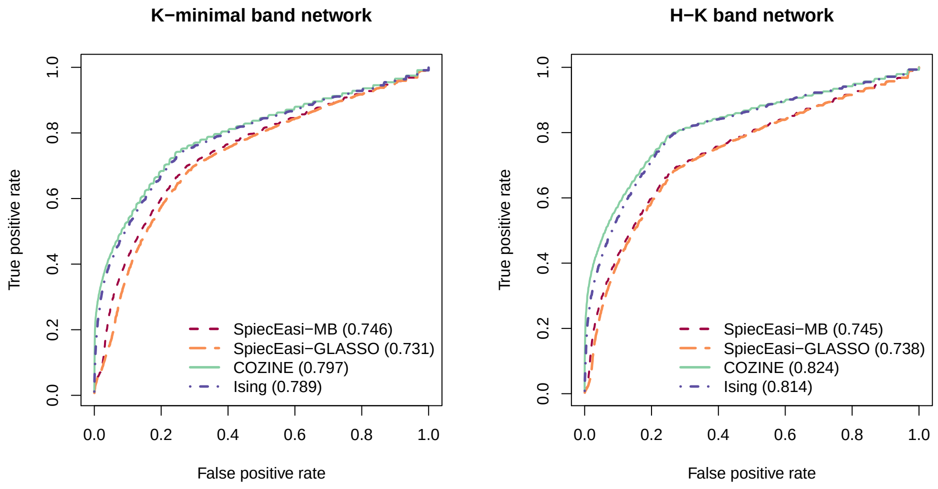

Figure S1: **Performance comparison on simulated data.** ROC curves and AUC values for SpiecEasi-MB, SpiecEasi-GLASSO, COZINE, and Ising model under the K-minimal and H-K band networks scenarios.

S1.2 High-dimensional networks

In order to compare the performance of COZINE with other methodologies in a high-dimensional and more highly zero inflated setting, we have added an additional simulation study on the *G-H-K* scale-free network where the number of nodes is $p = 1000$. The data generation follows the same procedure as for the low-dimensional settings, as described in detail the "Simulation study" section.

Our simulation study is based on 25 replicated datasets with a sample size of $n = 200$. These data have an average proportion of zero values of 0.75. COZINE showed the highest accuracy in estimating the true network structure, with an AUC value of 0.766 (Figure S2). The average MCC value for COZINE was 0.33, which was significantly higher than those from other three methods: 0.08, 0.05 and 0.04 for SpiecEasi-MB, SpiecEasi-GLASSO, and Ising model, respectively.

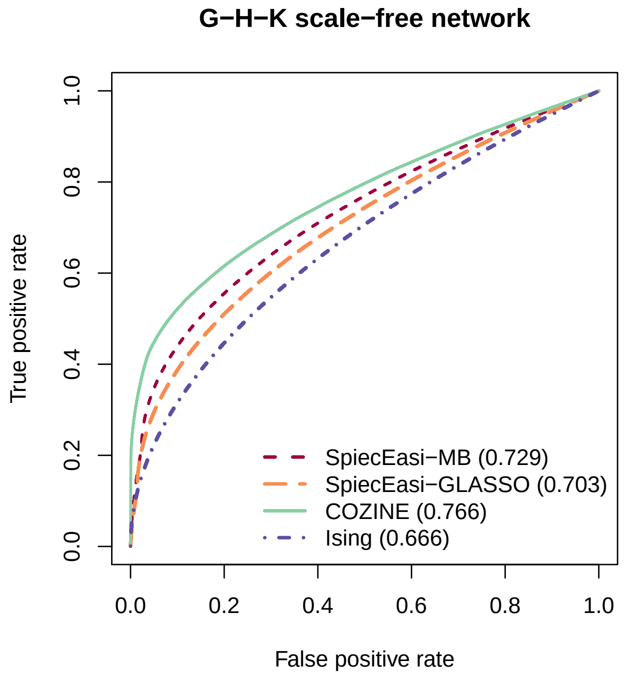

Figure S2: **Performance comparison on high-dimensional simulated data** ($p = 1000$ and $n = 200$) under *G-H-K* scale-free network. ROC curves and AUC values for SpiecEasi-MB, SpiecEasi-GLASSO, COZINE, and Ising model.

S2 Performance comparison on real microbiome data

Since the true microbial interactions are not known for the case study data, we evaluated performance by measuring the stability of the estimated networks. We compare 6 methods, including 4 partial correlation-based methods (COZINE, Ising, SpiecEasi-GLASSO and SpiecEasi-MB) and 2 additional correlation-based methods: SparCC (Friedman et al., 2008) and CCLasso (Fang et al., 2015). The stability of each edge is defined by the proportion of bootstrap samples where the resulting networks include the edge. To compute this, we randomly select $n = 86$ samples with replacement to estimate the network using all the 6 methods in comparison and then compute the stability value for each edge across 100 bootstrap samples.

For the additional marginal correlation-based models that do not provide sparse solutions, we considered an absolute correlation threshold of 0.35 and p-value cutoff of 0.00001 for SparCC and CCLasso, respectively, as used in Kurtz et al. (2015) in their comparisons. The estimated networks included 59 (COZINE), 117 (Ising), 61 (SpiecEasi-MB), 27 (SpiecEasi-GLASSO), 102 (SparCC) and 76 (CCLasso) edges. Figure S3 displays stability values across edges from the 6 different methods. COZINE showed the best performance in terms of stability with the highest median value of 0.84. Ising and SpiecEasi-GLASSO showed similar performance with median value of 0.82 and 0.81, respectively. However, the network from the Ising method includes edges that have a low stability of less than 0.5, and none of the edges from SpiecEasi-GLASSO achieved stability of 1. SpiecEasi-MB was sensitive to the perturbation in data with the lowest level of stability.

We next evaluate the performance in terms of assortativity to investigate the tendency of genera which occur in the same branch of the taxonomic tree to be linked within co-occurrence networks estimated from the 6 approaches (Table S1). COZINE showed the most significant assortative mixing overall, with highest coefficients at the Class and Order levels across the methods compared.

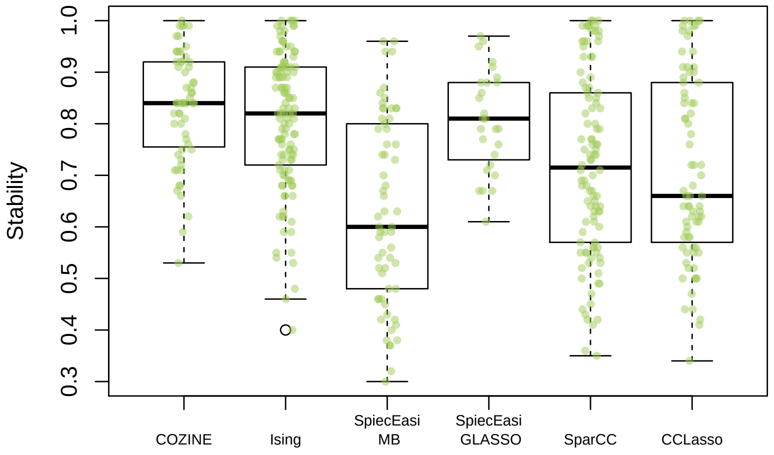

Figure S3: **Stability of edges in estimated networks**

Table S1: Assortativity coefficient (p-values from 100,000 permutations)

Level	COZINE	SpiecEasi-MB	SpiecEasi-GLASSO	Ising	SparCC	CCLasso
Phylum	0.26 (0.0001)	0.22 (0.0002)	0.29 (0.0013)	0.14 (0.0003)	0.18 (7e-05)	0.27 (1e-05)
Class	0.22 (< 1e-05)	0.16 (< 1e-05)	0.20 (3e-04)	0.06 (0.0019)	0.15 (< 1e-05)	0.17 (1e-05)
Order	0.15 (< 1e-05)	0.08 (0.0014)	0.11 (0.0029)	0.04 (0.0044)	0.07 (0.0002)	0.09 (6e-05)
Family	0.10 (< 1e-05)	0.10 (1e-05)	0.15 (1e-05)	0.06 (2e-05)	0.06 (< 1e-05)	0.09 (<1e-05)

S3 Inference of OTU network

We also applied COZINE to our case study data defined at the OTU level. The data include 2029 OTU counts and show a high level of sparsity, with a proportion of zero values of 95%. Our method took 3.03 hours on a Linux server (2.93 GHz, 96GB RAM). We found 3058 edges among 518 OTUs (vertices), which is 0.15% of all possible edges. Figure S4 displays the co-occurrence network at the OTU level, with the degree distribution of vertices as an inset. As seen from the degree distribution and the topological structure, the network shows the scale-free property with hubs that have a large number of edges. There were four prominent hub nodes that had degree greater than 100, listed in Table S2.

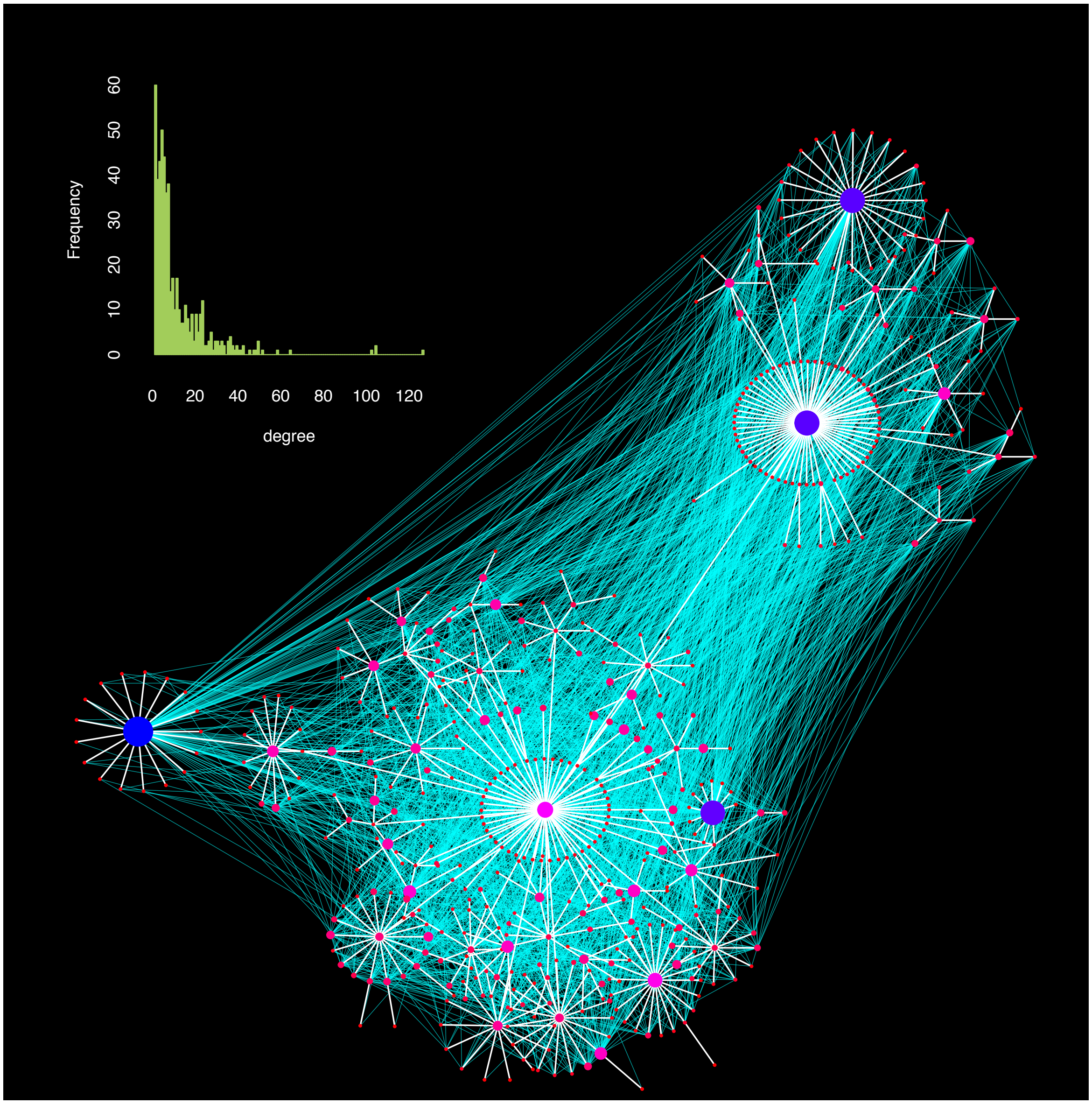

Figure S4: Co-occurrence network at the species level, with the corresponding degree distribution as an inset.

Table S2: Hub nodes that have degree >100

Kingdom	Phylum	Class	Order	Family	Genus	OTU	Degree
Bacteria	Verrucomicrobia	Verrucomicrobiae	Verrucomicrobiales	Verrucomicrobiaceae	Akkermansia	Unc00b5g	127
Bacteria	Proteobacteria	Gammaproteobacteria	Enterobacteriales	Enterobacteriaceae	Enterobacter	Unc93480	105
Bacteria	Bacteroidetes	Bacteroidia	Bacteroidales	Bacteroidaceae	Bacteroides	BctNL659	105
Bacteria	Firmicutes	Erysipelotrichia	Erysipelotrichales	Erysipelotrichaceae	IncertaeSedis	BctNL543	103

References

Fang, H., Huang, C., Zhao, H., and Deng, M. (2015). CCLasso: correlation inference for compositional data through Lasso. *Bioinformatics*, 31(19):3172–3180.

Friedman, J., Hastie, T., and Tibshirani, R. (2008). Sparse inverse covariance estimation with the graphical lasso. *Biostatistics*, 9(3):432–441.

Kurtz, Z. D., Müller, C. L., Miraldi, E. R., Littman, D. R., Blaser, M. J., and Bonneau, R. A. (2015). Sparse and compositionally robust inference of microbial ecological networks. *PLoS computational biology*, 11(5):e1004226.
